# Antimicrobial Susceptibility of Staphylococcus Isolates from the Skin of Patients with Diarrhea-Predominant Irritable Bowel Syndrome Treated with Repeat Courses of Rifaximin

**DOI:** 10.1128/AAC.02165-16

**Published:** 2016-12-27

**Authors:** Herbert L. DuPont, Ray A. Wolf, Robert J. Israel, Mark Pimentel

**Affiliations:** a The University of Texas School of Public Health, Kelsey Research Foundation, Baylor College of Medicine, Baylor St. Luke's Medical Center, Houston, Texas, USA; b Salix Pharmaceuticals, Raleigh, North Carolina, USA; c Cedars-Sinai Medical Center, Los Angeles, California, USA

**Keywords:** antibiotics, irritable bowel syndrome with diarrhea, resistance, rifaximin, skin swabs, staphylococcal isolates, susceptibility

## LETTER

Rifaximin is a nonsystemic antibiotic indicated for diarrhea-predominant irritable bowel syndrome (IBS-D) (1). A randomized, double-blind (DB), placebo-controlled, phase 3 trial (TARGET 3; NCT01543178) assessed up to three 2-week courses of rifaximin (2). Adults with IBS-D who responded to 2 weeks of open-label rifaximin treatment (550 mg three times a day [TID]) during a 4-week posttreatment follow-up but subsequently relapsed during 18 additional weeks of follow-up were randomly assigned to DB treatment with rifaximin or placebo for two 2-week repeat courses (10 weeks apart). Given the potential risk of antibiotic resistance, the antibiotic susceptibility of Staphylococcus skin isolates was tested during various study phases.

(These data were presented in part at the American College of Gastroenterology Annual Scientific Meeting and Postgraduate Course, 16 to 21 October 2015, Honolulu, HI.)

Details of patient population and study design were previously published (2). In the current substudy, isolates were cultured from skin swabs of the peri-anus, nostrils, forearms, and palms of hands at 5 occasions: start and end of open-label rifaximin treatment, start and end of first DB treatment, and study end. Cultures were analyzed at central laboratories. Skin swabs were plated on both tryptic soy agar with 5% sheep blood and Columbia colistin nalidixic acid (NCA) agar with 5% sheep blood and then incubated in a 5% to 7% CO_2_ incubator at 35°C for 24 and 48 h. Broth microdilution was used to determine MICs of 11 antibiotics—rifaximin, rifampin, ceftazidime, ceftriaxone, cephalothin, ciprofloxacin, imipenem, meropenem, piperacillin-tazobactam, trimethoprim-sulfamethoxazole, and vancomycin—against Staphylococcus isolates. MIC ranges were based on Clinical and Laboratory Standards Institute (CLSI) guidelines (3) or the literature (4). Plates containing each antibiotic were inoculated and incubated at 35°C ± 1°C in CO_2_. Purity control and positive- and negative-growth control plates were included. To provide side-by-side comparisons of rifaximin and rifampin, the rifampin MIC value for resistance (≥4 μg/ml) was assigned to rifaximin.

Skin swabs were obtained from 115 patients; 31 also participated in the DB phase (rifaximin treatment, *n* = 19; placebo treatment, *n* = 12). A total of 1,381 staphylococcal isolates (18 strains) were identified; the majority of the isolates were Staphylococcus epidermis (54.2%) or Staphylococcus hominis (17.2%) species. Staphylococcus haemolyticus (8.2%), Staphylococcus aureus (5.1%), and Staphylococcus capitis (4.3%) strains were less commonly isolated.

At the DB baseline, placebo group isolates had rifaximin MIC_50_ (0.015 μg/ml) and MIC_90_ (0.03 μg/ml) values identical to those observed with rifaximin (Table 1). Rifaximin MIC_50_ values remained low (0.015 to 0.06 μg/ml) through the end-of-study visit. Transient increases in rifaximin MIC_90_ values were observed in the DB-rifaximin group but not the DB-placebo group, with a return to DB baseline MIC_90_ values by the time of the end-of-study visit. Similar patterns in MIC_50_ and MIC_90_ values had been observed for rifaximin during open-label treatment (data not shown). Rifampin susceptibility results were comparable with rifaximin susceptibility results (DB data, Table 2). For other antibiotics tested, MIC values were also low, with minimal changes (DB data, Table 3). For the 71 S. aureus isolates, no rifaximin- or rifampin-resistant isolates were cultured, nor was there any S. aureus overgrowth apparent. Overall, this analysis of Staphylococcus skin isolates from patients with IBS-D demonstrated that short-term (2-week) exposure to rifaximin (1,650 mg/day for up to 3 courses) did not lead to clinically significant or persistent resistance to rifaximin, rifampin, or other clinically important antibiotics.

**TABLE 1:** *In vitro* activity of rifaximin and rifampin against Staphylococcus isolates obtained during the DB phase^*a*^

Time point^*b*^ (patients) and treatment group	No. of isolates	Rifaximin (μg/ml)			Rifampin (μg/ml)		
		MIC range	MIC_50_	MIC_90_	MIC range	MIC_50_	MIC_90_
DB rifaximin							
Day 1 (*n* = 18)	65	≤0.001 to 64	0.015	0.03	≤0.015 to >32	≤0.015	≤0.015
Wk 2; EOT (*n* = 18)	64	0.004 to 64	0.015	32	≤0.015 to >32	≤0.015	16
Wk 11–14 (*n* = 1)	5	0.008 to 64	0.06	64	≤0.015 to >32	0.03	>32
Wk 15–18 (*n* = 1)	3	0.015 to 0.5	0.015	0.5	≤0.015 to 0.25	≤0.015	0.25
Wk 19–22 (*n* = 10)	43	0.008 to 0.32	0.015	0.5	≤0.015 to >32	≤0.015	0.12
Wk ≥ 23 (*n* = 6)	28	0.004 to 64	0.015	0.06	≤0.015 to >32	≤0.015	≤0.015
DB placebo							
Day 1 (*n* = 12)	48	≤0.001 to 64	0.015	0.03	≤0.015 to 8	≤0.015	≤0.015
Wk 2 (EOT; *n* = 12)	63	≤0.001 to 64	0.015	0.03	≤0.015 to >32	≤0.015	≤0.015
Wk 15–18 (*n* = 1)	4	0.008 to 0.03	0.03	0.03	≤0.015 to ≤0.015	≤0.015	≤0.015
Wk 19–22 (*n* = 5)	27	0.004 to 0.03	0.015	0.03	≤0.015 to ≤0.015	≤0.015	≤0.015
Wk ≥ 23 (*n* = 6)	29	0.008 to 0.06	0.015	0.03	≤0.015 to 0.03	≤0.015	≤0.015

a Patients received open-label (OL) rifaximin (550 mg 3 times daily [TID]) for 2 weeks followed by a 4-week, treatment-free follow-up period to determine response. Responders to OL rifaximin who experienced symptom recurrence during an 18-week treatment-free follow-up period were randomly assigned, in a double-blind (DB) manner, to receive 2 repeat treatments of rifaximin (550 mg) or placebo TID for 2 weeks, with the courses separated by 10 weeks.

b The follow-up periods differed; therefore, the follow-up visits were grouped into 4-week periods to determine whether there was an effect of time on antibiotic susceptibility of staphylococcal isolates. Only data from weeks in which isolates were obtained are shown in the table. EOT, end of treatment.

**TABLE 2:** Rifaximin- and rifampin-resistant staphylococcal isolates obtained during the DB phase of study^*a*^

Time point^*c*^ (patients)	No. of isolates	No. of antibiotic-resistant isolates from indicated location									
		Rifaximin^*b*^					Rifampin				
		Arms^*d*^	Nostrils	Palms	Perianal	Total	Arms^*d*^	Nostrils	Palms	Perianal	Total
DB rifaximin											
Day 1 (*n* = 18)	65	1	0	0	1	2	1	0	0	1	2
Wk 2; EOT (*n* = 18)	64	0	0	0	12	12	0	0	0	11	11
Wk 11–14 (*n* = 1)	5	0	0	0	2	2	0	0	0	2	2
Wk 15–18 (*n* = 1)	3	0	0	0	0	0	0	0	0	0	0
Wk 19–22 (*n* = 10)	43	0	0	0	4	4	0	0	0	4	4
Wk ≥ 23 (*n* = 6)	28	0	0	0	2	2	0	0	0	2	2
DB placebo											
Day 1 (*n* = 12)	48	0	0	0	1	1	0	0	0	1	1
Wk 2 (EOT; *n* = 12)	63	0	0	0	2	2	0	0	0	2	2
Wk 11–14 (*n* = 0)	0	0	0	0	0	0	0	0	0	0	0
Wk 15–18 (*n* = 1)	4	0	0	0	0	0	0	0	0	0	0
Wk 19–22 (*n* = 5)	27	0	0	0	0	0	0	0	0	0	0
Wk ≥ 23 (*n* = 6)	29	0	0	0	0	0	0	0	0	0	0

a Patients received open-label (OL) rifaximin (550 mg 3 times daily [TID]) for 2 weeks followed by a 4-week, treatment-free follow-up period to determine response. Responders to OL rifaximin who experienced symptom recurrence during an 18-week treatment-free follow-up period were randomly assigned, in a double-blind (DB) manner, to receive 2 repeat treatments of rifaximin (550) mg or placebo TID for 2 weeks, with the courses separated by 10 weeks. EOT, end of treatment.

b To compare the levels of sensitivity of *Staphylococcus* isolates to rifaximin and rifampin, the Clinical and Laboratory Standards Institute-established MIC breakpoint for rifampin (i.e., resistance at MIC ≥ 4 μg/ml) was applied to rifaximin.

c The follow-up periods differed; therefore, the follow-up visits were grouped into 4-week periods to determine whether there was an effect of time on antibiotic susceptibility of staphylococcal isolates. Only data from weeks in which isolates were obtained are shown in the table.

d Data from forearms of each patient were pooled.

**TABLE 3:** *In vitro* activity^*a*^ of 9 antibiotics against staphylococcal isolates obtained during the DB phase^*b*^

Time point^*c*^ (patients)	MIC_50_ (μg/ml)								
	CAZ	CRO	CEF	CIP	IPM	MEM	TZP	SXT	VAN
DB rifaximin									
Day 1 (*n* = 18)	8	2	0.25	0.25	0.015	0.12	0.5/4	0.25/4.8	1
Wk 2; EOT (*n* = 18)	4	2	0.12	0.25	0.015	0.12	0.25/4	0.25/4.8	1
Wk 11–14 (*n* = 1)	16	4	0.25	0.5	0.015	0.12	0.5/4	0.25/4.8	1
Wk 15–18 (*n* = 1)	4	1	0.12	0.25	0.015	0.12	0.25/4	0.5/9.5	2
Wk 19–22 (*n* = 10)	8	2	0.12	0.25	0.015	0.12	0.5/4	0.25/4.8	1
Wk ≥ 23 (*n* = 6)	8	2	0.25	0.25	0.015	0.12	0.5/4	0.12/2.4	1
DB placebo									
Day 1 (*n* = 12)	8	2	0.25	0.25	0.015	0.12	0.5/4	0.25/4.8	1
Wk 2; EOT (*n* = 12)	8	2	0.25	0.25	0.015	0.12	0.5/4	0.25/4.8	1
Wk 15–18 (*n* = 1)	8	4	0.25	1	0.03	0.25	0.5/4	0.06/1.2	1
Wk 19–22 (*n* = 5)	8	2	0.12	0.25	0.015	0.12	0.5/4	0.25/4.8	1
Wk ≥ 23 (*n* = 6)	8	4	0.25	0.25	0.015	0.25	0.5/4	0.25/4.8	1

a Based on MIC_50_ values. CAZ, ceftazidime; CEF, cephalothin; CIP, ciprofloxacin; CRO, ceftriaxone; DB, double-blind; EOT, end of treatment; IPM, imipenem; MEM, meropenem; SXT, trimethoprim-sulfamethoxazole; TZP, piperacillin-tazobactam; VAN, vancomycin.

b Patients received open-label (OL) rifaximin (550 mg 3 times daily [TID]) for 2 weeks followed by a 4-week, treatment-free follow-up period to determine response. Responders to OL rifaximin who experienced symptom recurrence during an 18-week treatment-free follow-up period were randomly assigned, in a DB manner, to receive 2 repeat treatments of rifaximin (550 mg) or placebo TID for 2 weeks, with the courses separated by 10 weeks.

c The follow-up periods differed; therefore, the follow-up visits were grouped into 4-week periods to determine whether there was an effect of time on antibiotic susceptibility of staphylococcal isolates. Only data from weeks in which isolates were obtained are shown in the table.

(This study has been registered at ClinicalTrials.gov under registration no. NCT01543178.)
